# 696. Effectiveness of Oral Vancomycin as Prophylaxis against *Clostridioides difficile* Infection in Hematopoietic Stem Cell Transplant Patients

**DOI:** 10.1093/ofid/ofad500.758

**Published:** 2023-11-27

**Authors:** Kelly M Reitmeyer, Brijesh Rana, David A Awad, Esther Huang, Jiyeon J Park, Arsheena Yassin, John P Mills, Ahmed Abdul Azim, Pinki Bhatt, Navaneeth Narayanan

**Affiliations:** Robert Wood Johnson University Hospital, Forked River, New Jersey; Rutgers University, New Brunswick, New Jersey; Robert Wood Johnson University Hospital, Forked River, New Jersey; Rutgers Cancer Institute of New Jersey, New Brunswick, New Jersey; Englewood Health, Englewood, New Jersey; Robert Wood Johnson University Hospital, Forked River, New Jersey; Rutgers Robert Wood Johnson Medical School, Montclair, New Jersey; Rutgers Robert Wood Johnson Medical School, Montclair, New Jersey; Rutgers - Robert Wood Johnson Medical School, New Brunswick, New Jersey; Rutgers University Ernest Mario School of Pharmacy & Robert Wood Johnson University Hospital, New Brunswick, NJ

## Abstract

**Background:**

Patients receiving hematopoietic stem cell transplants (HSCT) are at increased risk for *Clostridioides difficile* infection (CDI) due to decreased immune function, frequent exposure to broad-spectrum antibiotics and chemotherapy, prolonged hospitalizations, and alteration in gut microbiome. The purpose of this study was to assess the effectiveness of oral vancomycin prophylaxis (OVP) for CDI in all patients admitted for HSCT.

**Methods:**

This was a single-center, retrospective, cohort study conducted at a tertiary care academic medical center in New Jersey. Medical records of patients admitted between June 2019 to August 2022 to undergo an allogeneic or autologous HSCT were reviewed. Universal OVP from admission to discharge for HSCT patients began December 2019. Patients ≥ 18 years at the time of admission for the HSCT were included. Patients who were admitted less than 72 hours or who were being treated for an active CDI prior to HSCT were excluded. The primary endpoint was the incidence of in-hospital CDI. Secondary endpoints included incidence of vancomycin-resistant enterococci (VRE) bloodstream infections, VRE isolated from any clinical culture, gram-negative bloodstream infections, hospital survival, and hospital length of stay. Exploratory endpoints including one-year survival, relapse, and incidence of graft-versus-host disease were also collected. Confounding by relevant covariates were assessed and controlled for in a multivariable regression model.

**Results:**

A total of 132 HSCT patients were included. There was one case (1/68, 1.47%) of CDI in the prophylaxis group compared to six CDI cases (6/64, 9.38%) in the no prophylaxis group (P=0.057). There were no significant (P>0.05) between-group differences of incidence of gram-negative bloodstream infections, hospital survival, and length of stay. There were zero clinical cultures positive for VRE in either group.

**Figure:**
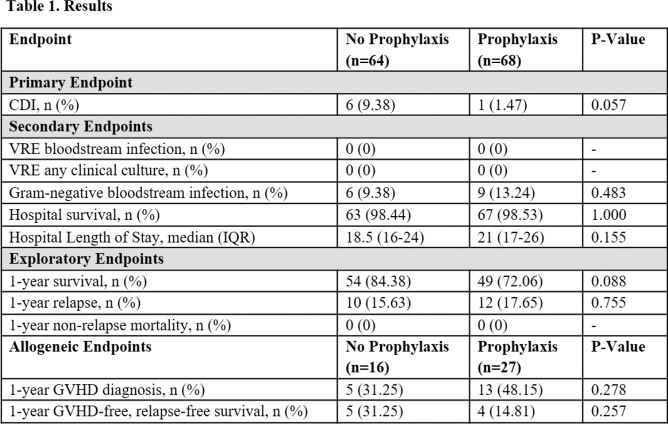

**Conclusion:**

In-hospital incidence of CDI in HSCT patients was decreased with the use of OVP but did not meet statistical significance. Randomized controlled trials are needed in this high-risk patient population to assess the efficacy and long-term risks of OVP for CDI.

**Disclosures:**

**Pinki Bhatt, MD**, Sanofi: Grant/Research Support **Navaneeth Narayanan, PharmD, MPH, BCIDP**, Astellas: Honoraria|Beckman Coulter: Honoraria|Merck: Grant/Research Support|Shionogi: Grant/Research Support

